# The association between personal hygiene and mental health, and the modifying roles of the universal health coverage index in children and adolescents: a comparative analysis from multiple countries

**DOI:** 10.7189/jogh.16.04184

**Published:** 2026-08-14

**Authors:** Yuwei Zhang, Hongzhi Zhou, Yi Liang, Xuanyu Shi, Zhi Li, Wenjie Wang, Xueling Wang

**Affiliations:** 1 Department of Stomatology, Aerospace Center Hospital, Beijing, China; 2 Institute of Medical Technology, Peking University, Beijing, China; 3 National Institute of Health Data Science, Peking University, Beijing, China

**Keywords:** personal hygiene, mental health, adolescents, universal health coverage, Global School-based Student Health Survey

## Abstract

**Background:**

Personal hygiene and mental health are both core determinants of adolescent well-being, yet their interplay and the modifying role of national health system coverage remain largely unexplored in cross-national populations. Therefore, we aimed to investigate associations between personal hygiene behaviours and mental health outcomes in adolescents and the modifying factors of the universal health coverage (UHC) index across multiple countries.

**Methods:**

We analysed data on 153,413 adolescents aged 12–17 from 51 countries collected through the Global School-based Student Health Survey between 2013 and 2019. Self-reported hygiene behaviours included toothbrushing and handwashing, and mental health outcomes included suicide ideation, bullying, being lonely, worry-related sleep disorders, and having no close friends. We examined associations using binary logistic regression models, stratified by sex and UHC index groups.

**Results:**

We found that all four hygiene behaviours were generally associated with improved mental health outcomes in boys and girls (*P* < 0.001). For boys, differences between UHC groups in hygiene scores were associated with mental health outcomes, particularly with suicide ideation (lower UHC: odds ratio (OR) = 0.88; 95% confidence interval (CI) = 0.85–0.91; higher UHC: OR = 0.82; 95% CI = 0.79–0.85), bullying (lower UHC: OR = 0.84; 95% CI = 0.82–0.86; higher UHC: OR = 0.78; 95% CI = 0.76–0.80), being lonely (lower UHC: OR = 0.91; 95% CI = 0.89–0.93; higher UHC: OR = 0.88; 95% CI = 0.86–0.90), and having no close friends (lower UHC: OR = 0.82; 95% CI = 0.79–0.85; higher UHC: OR = 0.77; 95% CI = 0.74–0.80). For girls, higher hygiene scores were only associated with lower odds of being lonely in UHC groups, with a significant difference between groups (lower UHC: OR = 0.88; 95% CI = 0.86–0.90; higher UHC: OR = 0.81; 95% CI = 0.79–0.83, *P*-value for difference ≤0.001).

**Conclusions:**

Our analysis indicates that individual hygiene practices are associated with reduced risks of adverse mental health outcomes in adolescents. The UHC index seems to play a modifying role in this context, with amplified associations in higher UHC coverage countries, particularly for boys.

Mental health is a critical element of overall well-being, yet it remains one of the most overlooked aspects of health. Children and adolescents, in particular, are vulnerable to mental health issues due to the rapid physical, emotional, and social changes they experience during this period of life [1–3]. However, in many areas, particularly in low- and middle-income countries (LMICs), where 90% of the world’s children live, mental health issues are often underdiagnosed and underreported [4]. This situation is mainly exacerbated by limited access to appropriate education and mental health resources, and a lack of awareness about the importance of mental well-being [5]. This neglect impairs the quality of life for young individuals, and also hinders their academic performance, social relationships, and future productivity [6]. Therefore, understanding the determinants of mental health in this demographic is essential for developing effective interventions.

Another fundamental aspect of health is personal hygiene, which is often a significant challenge in lower-income countries, where access to clean water, sanitation facilities, and health education is often limited [7], potentially affecting both physical health and psychological well-being [8,9]. For adolescents – a population particularly sensitive to social perceptions and peer relationships – poor hygiene may contribute to feelings of shame, social isolation, and low self-worth, potentially exacerbating mental health issues [10].

Although existing research has examined mental health and hygiene independently, few studies have investigated the intersection of these factors [11], particularly in the situation of adolescents from LMICs [12]. Furthermore, little is known about how the national level of health services quality, defined by universal health coverage (UHC), might modify the association between personal hygiene and mental health. To address these knowledge gaps, we utilised data from the Global School-based Student Health Surveys (GSHS) conducted in 51 countries between 2013 and 2019 to examine the association between individual hygiene habits and various mental health indicators, and to explore whether these associations differ by country-level UHC index. Our primary hypothesis is that better personal hygiene habits are associated with a lower risk of mental health problems, and we assume the associations would be greater in countries with higher UHC index levels.

## METHODS

### Study population and survey design

We analysed data from the rounds of the GSHS spanning from 2013 to the last available year [13,14]. The GSHS is a collaborative surveillance project aimed at helping countries measure and assess behavioural risk factors and protective factors among adolescents aged 12–17 across 10 key areas [15,16]. The survey employs a two-stage sampling design: in the first stage, schools are randomly selected with a probability proportional to the number of enrolled students, and in the second stage, classes within these schools are randomly selected using a systematic equal-probability sampling method. Data collection is carried out through self-administered questionnaires using standardised methodology [17,18]. The raw data from the GSHS are freely accessible on the World Health Organization's website [19]. Our study is a secondary analysis of the pre-existing, nationally representative GSHS data. As such, we did not perform a formal sample size calculation *a priori*. We selected the 2013–2019 period to ensure the use of standardised, contemporary data, capturing recent global trends in hygiene practices and mental health outcomes, as previous research has shown significant shifts in adolescent behaviours over the past decade [10]. We thus conducted a pooled analysis of data from three pre-existing surveys spanning from 2013 to 2019, all of which employed a cross-sectional design and utilised a similar stratified multistage sampling method.

However, the final sample of 153,413 adolescents from 51 countries was sufficiently large to evaluate the associations of interest, as reflected by the narrow confidence interval (CI) observed in our analyses.

### Exposure variables

The GSHS assessed hygiene-related behaviours among participants by examining several key exposures [20]. These included times of brushing teeth per day, washing hands before eating, washing hands after toilet, and washing hands with soap. We evaluated each of these behaviours based on self-reported data collected over the past 30 days. For toothbrushing, participants were asked how many times per day they usually cleaned or brushed their teeth. The responses were coded from 1 to 6 on an ordinal scale, with full response descriptions and coding specifications provided (**Online Supplementary Document**). This allowed for a detailed understanding of oral hygiene practices. Handwashing behaviours were assessed through three specific questions. Participants were asked how often they washed their hands before eating, after using the toilet or latrine, and how often they used soap when washing their hands. A composite hygiene score was constructed by summing the scores of the four hygiene behaviours, all coded from 0 to 1 with different options for frequency responses (**Online Supplementary Document**). The final score showed acceptable internal consistency (Cronbach’s alpha = 0.71).

### Outcome assessments

Our study evaluated several mental health outcomes to understand the psychological well-being of participants [21]. Participants were asked if they had seriously considered attempting suicide in the past 12 months. Five self-reported mental health outcomes (suicide ideation, bullying victimisation, loneliness, worry-related sleep disturbance, and lack of close friends) were assessed and dichotomised into binary yes/no variables, with full original response scales provided (**Online Supplementary Document**).

### Covariates

We accounted for several confounding factors in our analysis, selecting covariates based on previous studies of the association between health behaviours and mental health outcomes in adolescents [22,23]. We did not use stepwise selection or data-driven criteria when creating the final adjustment set, meaning that the final multivariable models included all the preselected covariates. These factors covered four core domains: individual demographic characteristics (*e.g.* age, body mass index (BMI)), lifestyle behaviours (*e.g.* alcohol use, tobacco use, physical activity, sedentary time), mental well-being indicators, and country-level sociodemographic indices. Full variable definitions, questionnaire items and classification criteria are presented in Table S1 (**Online Supplementary Document**).

Demographic characteristics encompassed self-reported age, sex, and BMI. We calculated BMI using self-reported height and weight at the time of the survey and was converted to z-scores based on the WHO growth reference. Lifestyle factors including alcohol use, tobacco use, physical activity, and sedentary behaviours were assessed as covariates. Full questionnaire items, response scales and classification criteria for all these variables are provided in Table S1 (**Online Supplementary Document**). Additionally, country-level sociodemographic status was represented by the Sociodemographic Index (SDI) for the corresponding survey year, an indicator of national social and economic development.

UHC is a central focus of the international health agenda and plays a crucial role in achieving the Sustainable Development Goals (SDGs). The country-specific UHC Index, based on the year of GSHS data collection, was obtained from the World Bank website [24]. The UHC Index target was reasserted in the United Nations General Assembly High-Level Meeting in 2019, based on which we divided it into two groups (higher group with UHC Index ≥60, *vs.* lower group with UHC Index <60) [25].

### Statistical analysis

The demographic characteristics of the study population were summarised using descriptive statistics. For continuous variables, means and standard deviations (SD) were reported, while frequencies (proportions) were provided for categorical variables. A bar chart was utilised to illustrate the distribution of individual hygiene behaviours and scores by sex. Participants with missing data for key exposure, outcome, or covariate variables were excluded using a complete-case analysis. This approach was chosen because the proportion of missing data for each variable was less than 5% and was considered to be missing at random.

Additionally, binary logistic regression models were employed to analyse the associations between individual hygiene behaviours and scores and mental health outcomes. These models were adjusted for the above-mentioned covariates. With the intraclass correlation coefficients (ICC) below 0.05, the influence of ‘country’ on the variance in outcomes is minimal, indicating that the ‘country’ variable should be adjusted as a fixed effect, without using a random effects model [26].

Stratified analyses by sex and UHC groups were conducted to further explore these associations. To assess trends, the Cochran-Armitage trend test was applied to analyse the association between individual hygiene scores and mental health outcomes, stratified by sex.

Several sensitivity analyses were conducted. First, we applied mixed effects models to estimate associations of individual hygiene behaviours and mental health outcomes. Second, the scatter plot was applied to fit the effects of each country between the UHC index and the odds of hygiene behaviours and mental health outcomes.

Statistical analyses were performed using *R*, version 4.3.1 (R Foundation for Statistical Computing, Vienna, Austria), with two-sided *P*-values <0.05 considered statistically significant.

## RESULTS

### Participant characteristics

The baseline characteristics of the 153,413 participants (mean age = 14.69 years, SD = 1.47) were compared between boys (n = 70,916) and girls (n = 82 497) (**Table 1**). The overall average BMI was 20.85, SD = 4.70, with boys having a slightly lower average BMI of 20.55, SD = 4.65, and girls showing a higher average BMI of 21.11, SD = 4.73. Additionally, the proportions of participants reporting suicide ideation, bullying, being lonely, and worry-related sleep disorders varied significantly between boys and girls (*P* < 0.001). Suicide ideation and being lonely were more common in girls (11.46% and 69.96%) than boys (9.00% and 60.03%), while bullying was more prevalent in boys (29.84%) compared to girls (25.55%). Similarly, worry-related sleep disorders were reported more frequently by girls (66.50%) than boys (56.38%). However, no close friends were more similar between boys (5.71%) and girls (5.76%), with no significant gender difference (*P* = 0.659). Meanwhile, there was a significant difference between boys and girls for the UHC group (*P* < 0.001).

**Table 1:** Baseline of participant characteristics*

Characteristics	Total (n = 153,413)	Boys (n = 70,916)	Girls (n = 82,497)	*P*-value
Height, m, x̄ (SD)	1.58 (0.10)	1.62 (0.11)	1.55 (0.08)	<0.001
Weight, kg, x̄ (SD)	52.62 (14.77)	54.30 (15.85)	51.18 (13.60)	<0.001
BMI, x̄ (SD)	20.85 (4.70)	20.55 (4.65)	21.11 (4.73)	<0.001
Age in years, x̄ (SD)	14.69 (1.47)	14.72 (1.46)	14.67 (1.47)	<0.001
Suicide ideation				<0.001
*No*	137,576 (89.68)	64,535 (91.00)	73,041 (88.54)	
*Yes*	15,837 (10.32)	6,381 (9.00)	9,456 (11.46)	
Bullying				<0.001
*No*	101,320 (72.48)	45,125 (70.16)	56,195 (74.45)	
*Yes*	38,472 (27.52)	19,191 (29.84)	19,281 (25.55)	
Being lonely				<0.001
*No*	52,705 (34.62)	28,108 (39.97)	24,597 (30.04)	
*Yes*	99,516 (65.38)	42,219 (60.03)	57,297 (69.96)	
Worry-related sleep disorder				<0.001
*No*	58,383 (38.18)	30,828 (43.62)	27,555 (33.50)	
*Yes*	94,539 (61.82)	39,842 (56.38)	54,697 (66.50)	
No close friends				0.659
*No*	143,520 (94.26)	66,284 (94.29)	77,236 (94.24)	
*Yes*	8,736 (5.74)	4,013 (5.71)	4,723 (5.76)	
UHC				<0.001
*Low*	75,201 (50.59)	33,827 (49.24)	41,374 (51.75)	
*High*	73,448 (49.41)	34,873 (50.76)	38,575 (48.25)	
Tobacco use during the past 30 days				<0.001
*0 days*	118,167 (86.72)	50,255 (80.34)	67,912 (92.14)	
*≥1 days*	18,097 (13.28)	12,301 (19.66)	5796 (7.86)	
Alcohol use during the past 30 days				<0.001
*0 days*	110,062 (83.90)	48,708 (81.33)	61,354 (86.06)	
*≥1 days*	21,123 (16.10)	11,185 (18.67)	9938 (13.94)	
Physical activities 60 min per day				<0.001
*0 days*	39,644(26.22)	16,461 (23.58)	23,183 (28.48)	
*1 days*	31,313 (20.71)	12,820 (18.36)	18,493 (22.72)	
*2 days*	20,619 (13.64)	8,817 (12.63)	11,802 (14.50)	
*3 days*	14,771 (9.77)	6,924 (9.92)	7,847 (9.64)	
*4 days*	8,478 (5.61)	4,383 (6.28)	4,095 (5.03)	
*5 days*	8,459 (5.59)	4,327 (6.20)	4,132 (5.08)	
*6 days*	4,042 (2.67)	2,270 (3.25)	1,772 (2.18)	
*7 days*	23,891 (15.80)	13,818 (19.79)	10,073 (12.38)	
Daily sedentary time per day				<0.001
*<1 h*	4,042 (2.67)	2270 (3.25)	1,772 (2.18)	
*less than 1 h per day*	23,891 (15.80)	13,818 (19.79)	10,073 (12.38)	
*1 to 2 h per day*	46,277 (30.66)	21,801 (31.32)	24,476 (30.10)	
*3 to 4 h per day*	28,878 (19.13)	13,299 (19.11)	15,579 (19.16)	
*5 to 6 h per day*	11,672 (7.73)	5,251 (7.54)	6,421 (7.90)	
*7 to 8 h per day*	4,475 (2.96)	1,921 (2.76)	2,554 (3.14)	
*more than 8 h per day*	9,931 (6.58)	4,262 (6.12)	5,669 (6.97)	

BMI – body mass index, SD – standard deviation, UHC – universal health coverage, x̄ – mean

*Presented as n (%) unless specified otherwise.

### Distribution of individual hygiene and scores

**Figure 1:**
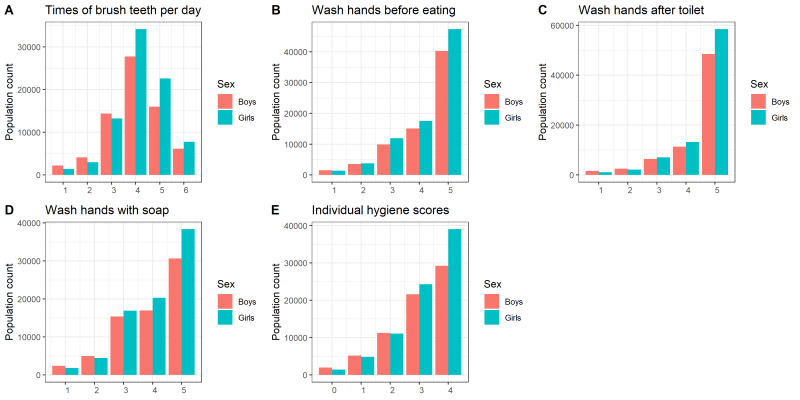
The distribution of individual hygiene behaviours and scores by sex. **Panel A.** The distribution of tooth brushing frequency among boys and girls; 1 = I did not clean or brush my teeth during the past 30 days; 2 = Less than 1 time per day; 3 = 1 time per day; 4 = 2 times per day; 5 = 3 times per day; 6 = 4 or more times per day. **Panel B.** The frequency of handwashing before eating; 1 = Never; 2 = Rarely; 3 = Sometimes; 4 = most of the time; 5 = Always. **Panel C.** The frequency of handwashing after toilet use; 1 = Never; 2 = Rarely; 3 = Sometimes; 4 = most of the time; 5 = Always. **Panel D.** The frequency of hand-washing with soap; 1 = Never; 2 = Rarely; 3 = Sometimes; 4 = most of the time; 5 = Always. **Panel E.** The distribution of individual hygiene scores; 0 = 0 score; 1 = 1 score; 2 = 2 scores; 3 = 3 scores; 4 = 4 scores.

The distribution of individual hygiene behaviours and scores differed by sex (**Figure 1**). Especially for individual hygiene scores, it ranged from 0 to 4, reflecting the hygiene conditions of boys and girls. In the category with a score of 0, there are 1,955 boys (2.82%) and 1,452 girls (1.80%). For a score of 1, there are 5,213 boys (7.53%) and 4,821 girls (5.97%). In the category with a score of 2, there are 11,230 boys (16.23%) and 11,093 girls (13.74%). For a score of 3, there are 21,600 boys (31.21%) and 24,314 girls (30.11%). Finally, in the category with a score of 4, there are 29,215 boys (42.21%) and 39,075 girls (48.39%). Overall, the hygiene scores for girls are generally higher than those for boys, particularly in the highest score category, where a larger proportion of girls have the highest score.

### Association between individual hygiene and mental health

Individual hygiene scores were significantly associated with mental health outcomes for both boys and girls in the adjusted model (**Table 2**). The four hygiene behaviours, brushing teeth per day, washing hands before eating, washing hands after toilet, and washing hands with soap were all significantly associated with improved mental health outcomes, showing reduced odds of suicide ideation, bullying, being lonely, worry-related sleep disorders, and having no close friends in both boys and girls (*P* < 0.001). Specifically, for boys, higher individual hygiene scores were associated with reduced odds of suicide ideation (odds ratio (OR) = 0.81; 95% CI = 0.80–0.83, *P* < 0.001), bullying (OR = 0.79; 95% CI = 0.78–0.81, *P* < 0.001), being lonely (OR = 0.85; 95% CI = 0.84–0.86, *P* < 0.001), worry-related sleep disorders (OR = 0.94; 95% CI = 0.92–0.95, *P* < 0.001), and having no close friends (OR = 0.80; 95% CI = 0.78–0.83, *P* < 0.001). Similarly, for girls, higher individual hygiene scores were also significantly associated with lower odds of suicide ideation (OR = 0.85; 95% CI = 0.83–0.88, *P* < 0.001), bullying (OR = 0.81; 95% CI = 0.80–0.83, *P* < 0.001), being lonely (OR = 0.87; 95% CI = 0.85–0.88, *P* < 0.001), worry-related sleep disorders (OR = 0.94; 95% CI = 0.92–0.95, *P* < 0.001), and having no close friends (OR = 0.83; 95% CI = 0.81–0.86, *P* < 0.001).

**Table 2:** Association between individual hygiene and mental health, stratified by sex

	Crude model		Adjusted model*	
**Individual hygiene and mental health**	**OR (95% CI)**	***P*-value**	**OR (95% CI)**	***P*-value**
**Boys**				
Times of brushing teeth per day				
*Suicide ideation*	0.91 (0.89–0.93)	<0.001	0.95 (0.93–0.98)	<0.001
*Bullying*	0.87 (0.86–0.88)	<0.001	0.93 (0.92–0.95)	<0.001
*Being lonely*	0.94 (0.93–0.95)	<0.001	0.93 (0.91–0.94)	<0.001
*Worry-related sleep disorder*	0.97 (0.96–0.98)	<0.001	0.97 (0.96–0.99)	<0.001
*No close friends*	0.85 (0.82–0.87)	<0.001	0.85 (0.83–0.88)	<0.001
Wash hands before eating				
*Suicide ideation*	0.80 (0.78–0.82)	<0.001	0.84 (0.82–0.86)	<0.001
*Bullying*	0.87 (0.85–0.88)	<0.001	0.85 (0.83–0.87)	<0.001
*Being lonely*	0.91 (0.90–0.93)	<0.001	0.91 (0.89–0.92)	<0.001
*Worry-related sleep disorder*	0.91 (0.90–0.93)	<0.001	0.91 (0.90–0.93)	<0.001
*No close friends*	0.81 (0.79–0.83)	<0.001	0.83 (0.80–0.85)	<0.001
Wash hands after toilet				
*Suicide ideation*	0.77 (0.75–0.78)	<0.001	0.80 (0.79–0.82)	<0.001
*Bullying*	0.79 (0.78–0.81)	<0.001	0.81 (0.80–0.83)	<0.001
*Being lonely*	0.91 (0.89–0.92)	<0.001	0.93 (0.92–0.95)	<0.001
*Worry-related sleep disorder*	0.94 (0.93–0.96)	<0.001	0.95 (0.93–0.96)	<0.001
*No close friends*	0.81 (0.78–0.83)	<0.001	0.80 (0.77–0.82)	<0.001
Wash hands with soap				
*Suicide ideation*	0.89 (0.87–0.91)	<0.001	0.91 (0.89–0.93)	<0.001
*Bullying*	0.88 (0.87–0.89)	<0.001	0.84 (0.83–0.86)	<0.001
*Being lonely*	0.89 (0.87–0.90)	<0.001	0.89 (0.88–0.91)	<0.001
*Worry-related sleep disorder*	0.92 (0.91–0.93)	<0.001	0.92 (0.91–0.94)	<0.001
*No close friends*	0.86 (0.83–0.88)	<0.001	0.86 (0.84–0.89)	<0.001
Individual hygiene scores				
*Suicide ideation*	0.79 (0.77–0.81)	<0.001	0.81 (0.80–0.83)	<0.001
*Bullying*	0.79 (0.78–0.81)	<0.001	0.79 (0.78–0.80)	<0.001
*Being lonely*	0.89 (0.88–0.90)	<0.001	0.85 (0.84–0.86)	<0.001
*Worry-related sleep disorder*	0.92 (0.91–0.94)	<0.001	0.94 (0.92–0.95)	<0.001
*No close friends*	0.78 (0.76–0.80)	<0.001	0.80 (0.78–0.83)	<0.001
**Girls**				
Times of brushing teeth per day				
*Suicide ideation*	0.96 (0.94–0.98)	<0.001	0.98 (0.95–1.00)	0.025
*Bullying*	0.90 (0.88–0.91)	<0.001	0.95 (0.93–0.97)	<0.001
*Being lonely*	0.96 (0.94–0.97)	<0.001	0.92 (0.90–0.93)	<0.001
*Worry-related sleep disorder*	0.99 (0.98–1.01)	0.360	0.99 (0.97–1.00)	0.064
*No close friends*	0.81 (0.79–0.84)	<0.001	0.86 (0.84–0.89)	<0.001
Wash hands before eating				
*Suicide ideation*	0.80 (0.78–0.82)	<0.001	0.87 (0.85–0.89)	<0.001
*Bullying*	0.87 (0.85–0.88)	<0.001	0.85 (0.83–0.86)	<0.001
*Being lonely*	0.85 (0.83–0.86)	<0.001	0.85 (0.84–0.87)	<0.001
*Worry-related sleep disorder*	0.89 (0.88–0.91)	<0.001	0.92 (0.91–0.94)	<0.001
*No close friends*	0.85 (0.83–0.88)	<0.001	0.86 (0.83–0.88)	<0.001
Wash hands after toilet				
*Suicide ideation*	0.82 (0.80–0.84)	<0.001	0.85 (0.83–0.87)	<0.001
*Bullying*	0.80 (0.79–0.82)	<0.001	0.82 (0.80–0.84)	<0.001
*Being lonely*	0.90 (0.88–0.92)	<0.001	0.91 (0.89–0.93)	<0.001
*Worry-related sleep disorder*	0.97 (0.95–0.99)	0.001	0.95 (0.94–0.97)	<0.001
*No close friends*	0.84 (0.81–0.86)	<0.001	0.83 (0.80–0.86)	<0.001
Wash hands with soap				
*Suicide ideation*	0.89 (0.87–0.91)	<0.001	0.92 (0.90–0.94)	<0.001
*Bullying*	0.87 (0.85–0.88)	<0.001	0.85 (0.83–0.86)	<0.001
*Being lonely*	0.86 (0.85–0.87)	<0.001	0.87 (0.85–0.88)	<0.001
*Worry-related sleep disorder*	0.94 (0.93–0.95)	<0.001	0.94 (0.93–0.96)	<0.001
*No close friends*	0.88 (0.86–0.91)	<0.001	0.87 (0.85–0.90)	<0.001
Individual hygiene scores				
*Suicide ideation*	0.86 (0.83–0.88)	<0.001	0.87 (0.86–0.89)	<0.001
*Bullying*	0.81 (0.80–0.83)	<0.001	0.81 (0.80–0.83)	<0.001
*Being lonely*	0.90 (0.88–0.91)	<0.001	0.85 (0.83–0.86)	<0.001
*Worry-related sleep disorder*	0.93 (0.92–0.94)	<0.001	0.94 (0.92–0.95)	<0.001
*No close friends*	0.80 (0.77–0.82)	<0.001	0.83 (0.81–0.86)	<0.001

CI – confidence interval, OR – odds ratio

*Model was adjusted for age, body mass index, countries, alcohol use, tobacco use, physical activities, sedentary behaviours, sociodemographic index, and study weight.

### Association between individual hygiene scores and mental health

The association between individual hygiene scores and mental health outcomes was examined, stratified by sex (Table S2 in the **Online Supplementary Document**). For boys, hygiene scores showed a significant negative association with suicide ideation, bullying, and no close friends, with higher hygiene scores associated with lower odds of these (*P*-value for trends <0.001). However, we did not find a statistically significant association between a hygiene score of 3 and loneliness (*P* > 0.05). A statistically significant association with worry-related sleep disorder was only found when the hygiene score was 4 (OR = 0.85; 95% CI = 0.77–0.94). For girls, as the hygiene score increases, the odds of mental health showed a downward trend (*P*-value for trends <0.001). Specifically, our study did not find a statistically significant association between scores of 3 or 4 and worry-related sleep disorder (*P* > 0.05).

### Association between individual hygiene and mental health across UHC

The association between individual hygiene scores and mental health outcomes was examined, stratified by sex and UHC group (**Table 3**). For boys, higher hygiene scores were associated with lower odds of suicide ideation in UHC groups (lower UHC: OR = 0.88; 95% CI = 0.85–0.91; higher UHC: OR = 0.82; 95% CI = 0.79–0.85, *P*-value for difference = 0.003). Hygiene scores showed a significant negative association with bullying, with lower odds of bullying in UHC groups (lower UHC: OR = 0.84; 95% CI = 0.82–0.86; higher UHC: OR = 0.78; 95% CI = 0.76–0.80, *P*-value for difference <0.001). Higher hygiene scores were associated with a decreased likelihood of being lonely in UHC groups (lower UHC: OR = 0.91; 95% CI = 0.89–0.93; higher UHC: OR = 0.88; 95% CI = 0.86–0.90, *P*-value for difference = 0.025). Hygiene scores were significantly associated with a lower likelihood of having no close friends, in UHC groups (lower UHC: OR = 0.82; 95% CI = 0.79–0.85; higher UHC: OR = 0.77; 95% CI = 0.74–0.80, *P*-value for difference = 0.026). However, hygiene scores showed a significant negative association with worry-related sleep disorders, with no significant difference between the UHC groups (*P*-value for difference = 0.990). For girls, we only found that higher hygiene scores were associated with lower odds of being lonely in both UHC groups, with a significant difference between the groups (lower UHC: OR = 0.88; 95% CI = 0.86–0.90; higher UHC: OR = 0.81; 95% CI = 0.79–0.83, *P*-value for difference = 0.026).

**Table 3:** Association between individual hygiene scores and mental health, stratified by sex and UHC group

	Lower UHC*		Higher UHC*		
**Mental health**	**OR (95% CI)**	***P*-value**	**OR (95%CI)**	***P*-value**	***P* for difference**
Boys					
*Suicide ideation*	0.88 (0.85–0.91)	<0.001	0.82 (0.79–0.85)	<0.001	0.003
*Bullying*	0.84 (0.82–0.86)	<0.001	0.78 (0.76–0.80)	<0.001	<0.001
*Being lonely*	0.91 (0.89–0.93)	<0.001	0.88 (0.86–0.90)	<0.001	0.025
*Worry-related sleep disorder*	0.93 (0.91–0.95)	<0.001	0.93 (0.91–0.95)	<0.001	0.990
*No close friends*	0.82 (0.79–0.85)	<0.001	0.77 (0.74–0.80)	<0.001	0.026
Girls					
*Suicide ideation*	0.88 (0.86–0.91)	<0.001	0.87 (0.84–0.90)	<0.001	0.465
*Bullying*	0.82 (0.80–0.84)	<0.001	0.79 (0.77–0.82)	<0.001	0.056
*Being lonely*	0.88 (0.86–0.90)	<0.001	0.81 (0.79–0.83)	<0.001	<0.001
*Worry-related sleep disorder*	0.93 (0.91–0.95)	<0.001	0.95 (0.92–0.97)	<0.001	0.461
*No close friends*	0.85 (0.82–0.89)	<0.001	0.81 (0.77–0.84)	<0.001	0.066

CI – confidence interval, OR – odds ratio, UHC – universal health coverage

*Model was adjusted for age, body mass index, countries, alcohol use, tobacco use, physical activities, sedentary behaviours, sociodemographic index, and study weight.

### Sensitivity analyses

The sensitivity analysis, stratified by sex and conducted using a mixed-effects model, reinforces the findings of the primary analysis. Among boys and girls, various individual hygiene behaviours were significantly and inversely associated with all measured mental health outcomes (Table S3 in the **Online Supplementary Document**). To further test the robustness of our findings, a sensitivity analysis was conducted to explore the potential moderating effect of UHC index. Scatter plots illustrated the association between the UHC index and the ORs for personal hygiene and various mental health outcomes (Figures S1–5 in the **Online Supplementary Document**). The visual evidence from the analysis indicated that the protective effect of personal hygiene on mental health was relatively stable across different levels of UHC index.

## DISCUSSION

In this large cross-sectional study of 153,413 adolescents from 51 countries, we examined personal hygiene habits and comprehensively assessed their associations with multiple mental health elements. We also explored the role of national health service accessibility, as measured by the UHC Index. Utilising the latest data from the GSHS, we found that the majority of adolescents in these countries maintained good oral and handwashing hygiene practices, with girls generally exhibiting better overall hygiene habits compared to boys. Better hygiene practices were associated with reduced risk of various mental health challenges, including suicide ideation, feelings of worry and loneliness, interpersonal problems, and worry-related sleep disturbance, in both boys and girls. The associations were particularly pronounced in countries with higher UHC Index. Given that both personal hygiene and mental health are often underprioritized in LMICs, our findings offer critical and practical insights into the complex interplay between individual behaviours, systemic health infrastructure, and mental well-being, highlighting potential avenues for targeted public health interventions, especially in resource-limited settings [3,27].

Over the past decades, Water, sanitation and hygiene (WASH) have contributed significantly to advances in global health [28,29]. Comprehensive, multicomponent theory-based hygiene promotion programs have been showing optimistic improvements on adolescents’ overall health worldwide [30,31]. Despite growing recognition of the importance of personal hygiene and mental health in adolescent well-being, data from multi-country studies examining their interplay remain scarce in LMICs. A cross-sectional study conducted between 2018 and 2019 in southwestern Nigeria reported that suicidal ideation was associated with a significantly lower likelihood of tooth brushing (OR = 0.48; 95% CI = 0.26–0.91) for adolescents [32]. Another study in Malawi also reported that mental health interventions could help improve hygiene practices in caregivers, suggesting a mutual reinforcement between these two factors [33]. Besides, harmonised, cross-national data on hygiene practice and mental health are still limited. Our current study, based on cross-sectionally comparable data from more than 50 countries, reports for the first time a positive and trending association between good personal hygiene practices and multiple mental health indicators, and finds that this association is more pronounced in countries with higher levels of the UHC index.

The observed associations between hygiene practices and mental health may be mediated by both biological and psychosocial pathways. Poor hygiene can increase susceptibility to infections, which are linked to systemic inflammation – a known risk factor for depression and anxiety [34]. For instance, chronic inflammation from untreated oral infections may disrupt neurotransmitter function, exacerbating mood disorders [35]. Additionally, hygiene practices may foster self-efficacy and routine-building, which are protective factors for mental health. Regular self-care rituals, such as brushing teeth or handwashing, can instil a sense of control and accomplishment, counteracting feelings of helplessness common in depression [36]. Psychosocially, adolescents with poor hygiene may face social stigma or bullying, amplifying loneliness and interpersonal conflicts [37]. This aligns with our findings that better hygiene correlates with reduced social problems. The stronger association in high-UHC countries suggests that accessible health services may amplify these benefits by integrating hygiene education with mental health support. For example, school-based programs in high-UHC settings often combine hygiene promotion with counselling, creating synergistic effects [38]. Future interventions could leverage this interplay by embedding mental health literacy within WASH initiatives, particularly in LMICs where fragmented health systems hinder holistic care.

The observed gender differences in hygiene practices and their associations with mental health warrant cautious interpretation. While girls in our sample reported consistently better hygiene practices than boys, a finding that aligns with previous research suggesting sociocultural norms around cleanliness may differ by gender [39,40], it is important to note that our data set does not contain measures of gender norms, stigma, or gendered expectations. Therefore, the mechanisms underlying these differences remain speculative. We propose that future research specifically measure these constructs to test hypotheses about how gender norms may shape both hygiene behaviours and their mental health implications. Our findings should be considered hypothesis-generating in this regard, rather than explanatory. However, this gendered dynamic could also explain why mental health benefits of hygiene are slightly more pronounced in girls, as adherence to socially validated behaviours may enhance self-esteem [41]. Conversely, boys with poor hygiene may experience compounded stigma, contributing to externalising behaviours like aggression rather than internalising symptoms [42]. Our findings also raise questions about the role of UHC in mitigating gender gaps. In countries with robust health systems, gender-sensitive hygiene campaigns – such as providing menstrual hygiene resources alongside mental health screenings – may address intersecting vulnerabilities [43]. Despite current progress, universal access to safe water, sanitation and hygiene by 2030 (as a remit of Sustainable Development Goal 6) remains a distant prospect in many countries, particularly in slums. Literature on personal hygiene and mental health surveys in such areas and other urban settings remains sparse [44,45].

These findings suggest that promoting good hygiene practices may offer a low-cost, scalable entry point for mental health promotion, particularly in resource-limited settings. For clinical practice, paediatricians and school health providers could consider assessing hygiene habits as part of routine mental health screening. For policy-making, Ministries of Health and Education should consider integrating mental health literacy into existing school-based WASH programs. This integrated approach could be particularly effective in low-UHC settings where mental health services are scarce, by leveraging a socially acceptable behaviour (hygiene) to address a highly stigmatised issue (mental health). Future interventions should be culturally adapted to account for cross-cultural differences in hygiene norms and health system structures.

The strength of this study is that it includes a large number of adolescents from more than 50 countries, all of whom were drawn from nationally representative samples. All of the included countries used a standardised scientific sample selection process, unified school-based methodology and questionnaires, and therefore facilitate valid assessments of cross-national or regional differences in hygiene practice-related mental health problems. However, some limitations of this study should be noted. First, the GSHS is a cross-sectional survey and is not conducted at the exact same time in each country, with the 51 countries included in this study having been researched from 2013 to the present. A primary limitation of this study is its cross-sectional design, which precludes any causal inference [15,16]. Reverse causality suggests that while poor hygiene may contribute to adverse mental health outcomes, it is equally plausible that poor mental health, as characterised by reduced motivation, self-care, and social functioning, may also lead to neglect of personal hygiene practices. The observed associations should therefore be interpreted as correlational, not causal. Moreover, although all the included countries used the standardised GSHS questionnaire for data collection, it was still possible that the data collection could be influenced by country-specific social and cultural circumstances. Second, all the data used in the present study relied on self-reported questionnaires. The reported prevalence and associations are inevitably subject to recall bias and underreporting due to the stigmatising effects of personal hygiene and mental health issues. Further qualitative research is needed to contextualise how hygiene influences mental well-being across genders and regions, and longitudinal studies tracking hygiene behaviour changes and mental health outcomes, alongside underlying biological mechanisms. Third, in the main analysis, report adjusted and unadjusted risk differences as ORs may reflect changes in covariate distributions rather than underlying risk relationships [46]. This difference (between a conditional and marginal odds/hazard ratio) is not explained by sampling variation, and is what is referred to when it is said that odds/hazard ratios are non-collapsible. Fourth, given the number of stratified analyses performed, the possibility of type I error due to multiple testing cannot be excluded. However, the consistent pattern and direction of associations across different outcomes, subgroups, and the highly significant *P*-values, typically <0.001, suggest that the main findings are unlikely to be solely attributable to chance. No formal adjustment for multiple comparisons was made, as this was considered an exploratory study aiming to generate hypotheses for further investigation. Fifth, despite adjusting for key individual- and country-level confounders, residual confounding remains a concern. The GSHS data set does not include information on potentially important structural determinants such as household deprivation, parental education and occupation, family structure, school environment, or household-level access to clean water and sanitation. These factors are likely associated with both hygiene practices and mental health outcomes, meaning the observed associations may partially reflect broader social disadvantage rather than a hygiene-specific effect. Sixth, dichotomisation of ordinal outcomes, while facilitating clinical interpretability, may result in a loss of granular information. However, this approach is commonly used in public health research to identify individuals with clinically meaningful levels of distress. Finally, no formal *a priori* sample size calculation was performed for this secondary analysis of pre-existing data, which constitutes a limitation of the study.

## CONCLUSIONS

In summary, this study demonstrates that better personal hygiene habits are significantly associated with reduced risks of poor mental health outcomes in children and adolescents across 51 countries. While these findings highlight a potentially important intersection between hygiene practices and mental well-being, the cross-sectional design precludes causal inference. Further longitudinal and interventional research is needed to determine the directionality and mechanisms of these associations. Nonetheless, our results suggest that hygiene and mental health may be intertwined in ways that merit consideration in integrated health promotion strategies.

## Additional material


Online Supplementary Document

## Data Availability

**Data availability:** All data generated or analysed during this study are included in this published article and its supplementary information files. The original data are available from the corresponding author upon reasonable request.
