# Supplementary material for: The association between personal hygiene and mental health, and the modifying roles of the universal health coverage index in children and adolescents: a comparative analysis from multiple countries
Source: J Glob Health. 2026 Aug 14;16:04184. doi: 10.7189/jogh.16.04184 (PMC13474066; doi:10.7189/jogh.16.04184)
Supplement: Supplementary file 1 [file jogh-16-04184-s001.pdf]

Supplement to: Zhang Y, Zhou H, Liang Y. The association between personal hygiene and mental health, and the modifying roles of the universal health coverage index in children and adolescents: a comparative analysis from multiple countries. J Glob Health. 2026;16:04184.

Table S1. Questionnaire details and definitions of exposures and outcomes.

Table S2. Association between individual hygiene scores and mental health, stratified by sex.

Table S3. Association between individual hygiene and mental health, stratified by sex using mixed-effects model.

Figure S1. The scatter plot between UHC index and the odds of times of brush teeth per day and mental health outcomes.

Figure S2. The scatter plot between UHC index and the odds of wash hands before eating and mental health outcomes.

Figure S3. The scatter plot between UHC index and the odds of wash hands after toilet and mental health outcomes.

Figure S4. The scatter plot between UHC index and the odds of wash hands with soap and mental health outcomes.

Figure S5. The scatter plot between UHC index and the odds of individual hygiene scores and mental health outcomes.

Table S1. Questionnaire details and definitions of exposures and outcomes for each of the datasets.

Variables	Questionnaire information	Classification criteria
	Exposures	
Times of brush teeth per day	During the past 30 days, how many times per day did you usually clean or brush your teeth?	1 = I did not clean or brush my teeth during the past 30 days; 2 = Less than 1 time per day; 3 = 1 time per day; 4 = 2 times per day; 5 = 3 times per day; 6 = 4 or more times per day.
Wash hands before eating	During the past 30 days, how often did you wash your hands before eating?	1 = Never; 2 = Rarely; 3 = Sometimes; 4 = Most of the time; 5 = Always
Wash hands after toilet	During the past 30 days, how often did you wash your hands after using the toilet or latrine?	1 = Never; 2 = Rarely; 3 = Sometimes; 4 = Most of the time; 5 = Always
Wash hands with soap	During the past 30 days, how often did you use soap when washing your hands?	1 = Never; 2 = Rarely; 3 = Sometimes; 4 = Most of the time; 5 = Always
	Outcome	
Suicide ideation	Participants were asked if they had seriously considered attempting suicide in the past 12 months?	1 = 0 times; 2 = 1 time; 3 = 2 or 3 times; 4 = 4 or 5 times; 5 = 6 or more times
Bullying	During the past 30 days, on how many days were you bullied?	1 = 0 days; 2 = 1 or 2 days; 3 = 3 to 5 days; 4 = 6 to 9 days; 5 = 10 to 19 days; 6 = 20 to 29 days; 7 = All 30 days
Being lonely	During the past 12 months, how often have you felt lonely?	1 = Never; 2 = Rarely; 3 = Sometimes; 4 = Most of the time; 5 = Always
Worry-related sleep disorder	During the past 12 months, how often have you been so worried about something that you could not sleep at night?	1 = Never; 2 = Rarely; 3 = Sometimes; 4 = Most of the time; 5 = Always
No close friends	How many close friends do you have?	1 = 0; 2 = 1; 3 = 2; 4 = 3 or more
	Covariates	
Alcohol use	During the past 30 days, on how many days did you have at least one drink containing alcohol?	1 = 0 days; 2 = 1 or 2 days; 3 = 3 to 5 days; 4 = 6 to 9 days; 5 = 10 to 19 days; 6 = 20 to 29 days; 7 = All 30 days
Tobacco use	During the past 30 days, on how many days did you smoke cigarettes?	1 = 0 days; 2 = 1 or 2 days; 3 = 3 to 5 days; 4 = 6 to 9 days; 5 = 10 to 19 days; 6 = 20 to 29 days; 7 = All 30 days
Physics activities	During the past 7 days, on how many days were you physically active for a total of at least 60 minutes per day?	1 = 0 days; 2 = 1 day; 3 = 2 days; 4 = 3 days; 5 = 4 days; 6 = 5 days; 7 = 6 days; 8 = 7 days
Sitting activities	How much time do you spend during a typical or usual day sitting and watching television, playing computer games, talking with friends, or doing other sitting activities such as playing cards?	1 = Less than 1 hour per day; 2 = 1 to 2 hours per day; 3 = 3 to 4 hours per day; 4 = 5 to 6 hours per day; 5 = 7 to 8 hours per day; 6 = More than 8 hours per day

Table S2. Association between individual hygiene scores and mental health, stratified by sex.

Individual hygiene scores in relation to mental health	Boys*		Girls*	
	OR (95% CI)	*P* value	OR (95% CI)	*P* value
Suicide ideation				
0	ref		ref	
1	0.66 (0.57–0.76)	<0.001	0.90 (0.77–1.06)	0.211
2	0.53 (0.46–0.60)	<0.001	0.73 (0.63–0.85)	<0.001
3	0.45 (0.39–0.51)	<0.001	0.64 (0.56–0.74)	<0.001
4	0.44 (0.39–0.50)	<0.001	0.58 (0.51–0.67)	<0.001
P for trend	<0.001		<0.001	
Bullying				
0	ref		ref	
1	0.77 (0.69–0.87)	<0.001	0.94 (0.82–1.07)	0.340
2	0.62 (0.55–0.69)	<0.001	0.72 (0.64–0.82)	<0.001
3	0.54 (0.48–0.60)	<0.001	0.61 (0.54–0.69)	<0.001
4	0.42 (0.37–0.46)	<0.001	0.48 (0.42–0.54)	<0.001
P for trend	<0.001		<0.001	
Being lonely				
0	ref		ref	
1	1.14 (1.02–1.28)	0.025	1.24 (1.08–1.43)	0.002
2	1.12 (1.01–1.24)	0.035	1.25 (1.10–1.42)	0.001
3	0.97 (0.88–1.08)	0.601	1.01 (0.89–1.15)	0.834
4	0.82 (0.74–0.90)	<0.001	0.79 (0.70–0.89)	<0.001
P for trend	<0.001		<0.001	
Worry-related sleep disorder				
0	ref		ref	
1	1.07 (0.96–1.20)	0.226	1.18 (1.03–1.34)	0.016
2	1.04 (0.94–1.15)	0.486	1.15 (1.02–1.30)	0.024
3	0.96 (0.87–1.06)	0.403	1.06 (0.94–1.20)	0.313
4	0.85 (0.77–0.94)	0.002	0.96 (0.86–1.08)	0.529
P for trend	<0.001		<0.001	
No close friends				
0	ref		ref	
1	0.56 (0.47–0.66)	<0.001	0.67 (0.55–0.81)	<0.001
2	0.47 (0.40–0.55)	<0.001	0.60 (0.50–0.72)	<0.001
3	0.39 (0.33–0.45)	<0.001	0.49 (0.41–0.58)	<0.001
4	0.32 (0.28–0.38)	<0.001	0.42 (0.35–0.50)	<0.001
P for trend	<0.001		<0.001	

CI – confidence interval, OR – odds ratio, ref – reference

*Model was adjusted for age, body mass index, countries, alcohol use, tobacco use, physical activities, sitting activities, sociodemographic index, and study weight.

Table S3. Association between individual hygiene and mental health, stratified by sex using mixed-effects model.

Individual hygiene	Mental health	Adjusted model*	
		OR (95% CI)	*P* value
	Boys		
Times of brush teeth per day	Suicide ideation	0.95 (0.93–0.98)	<0.001
	Bullying	0.93 (0.92–0.95)	<0.001
	Being lonely	0.93 (0.92–0.94)	<0.001
	Worry-related sleep disorder	0.98 (0.96–0.99)	0.001
	No close friends	0.85 (0.83–0.88)	<0.001
Wash hands before eating	Suicide ideation	0.83 (0.81–0.86)	<0.001
	Bullying	0.85 (0.83–0.87)	<0.001
	Being lonely	0.91 (0.89–0.92)	<0.001
	Worry-related sleep disorder	0.91 (0.90–0.93)	<0.001
	No close friends	0.83 (0.80–0.85)	<0.001
Wash hands after toilet	Suicide ideation	0.80 (0.78–0.82)	<0.001
	Bullying	0.81 (0.80–0.83)	<0.001
	Being lonely	0.93 (0.91–0.95)	<0.001
	Worry-related sleep disorder	0.95 (0.93–0.96)	<0.001
	No close friends	0.80 (0.77–0.82)	<0.001
Wash hands with soap	Suicide ideation	0.80 (0.78–0.82)	<0.001
	Bullying	0.91 (0.89–0.93)	<0.001
	Being lonely	0.84 (0.83–0.86)	<0.001
	Worry-related sleep disorder	0.89 (0.88–0.91)	<0.001
	No close friends	0.92 (0.91–0.94)	<0.001
Individual hygiene scores	Suicide ideation	0.86 (0.84–0.89)	<0.001
	Bullying	0.85 (0.83–0.87)	<0.001
	Being lonely	0.81 (0.80–0.83)	<0.001
	Worry-related sleep disorder	0.90 (0.88–0.91)	<0.001
	No close friends	0.93 (0.92–0.94)	<0.001
	Girls		
Times of brush teeth per day	Suicide ideation	0.98 (0.95–1.00)	0.031
	Bullying	0.95 (0.93–0.97)	<0.001
	Being lonely	0.92 (0.90–0.93)	<0.001
	Worry-related sleep disorder	0.99 (0.97–1.00)	0.084
	No close friends	0.86 (0.84–0.89)	<0.001
Wash hands before eating	Suicide ideation	0.87 (0.85–0.89)	<0.001
	Bullying	0.85 (0.83–0.86)	<0.001
	Being lonely	0.85 (0.84–0.87)	<0.001
	Worry-related sleep disorder	0.92 (0.91–0.94)	<0.001
	No close friends	0.86 (0.83–0.88)	<0.001
Wash hands after toilet	Suicide ideation	0.85 (0.83–0.87)	<0.001
	Bullying	0.82 (0.80–0.84)	<0.001
	Being lonely	0.91 (0.89–0.93)	<0.001
	Worry-related sleep disorder	0.95 (0.94–0.97)	<0.001
	No close friends	0.83 (0.80–0.86)	<0.001
Wash hands with soap	Suicide ideation	0.92 (0.90–0.94)	<0.001
	Bullying	0.85 (0.83–0.86)	<0.001
	Being lonely	0.87 (0.85–0.88)	<0.001
	Worry-related sleep disorder	0.94 (0.93–0.96)	<0.001
	No close friends	0.87 (0.85–0.90)	<0.001
Individual hygiene scores	Suicide ideation	0.87 (0.85–0.89)	<0.001
	Bullying	0.81 (0.80–0.83)	<0.001
	Being lonely	0.85 (0.83–0.86)	<0.001
	Worry-related sleep disorder	0.94 (0.92–0.95)	<0.001
	No close friends	0.83 (0.81–0.86)	<0.001

CI – confidence interval, OR – odds ratio.

*Model was adjusted for age, body mass index, alcohol use, tobacco use, physical activities, sitting activities, sociodemographic index, and study weight (countries was adjusted as random effects).

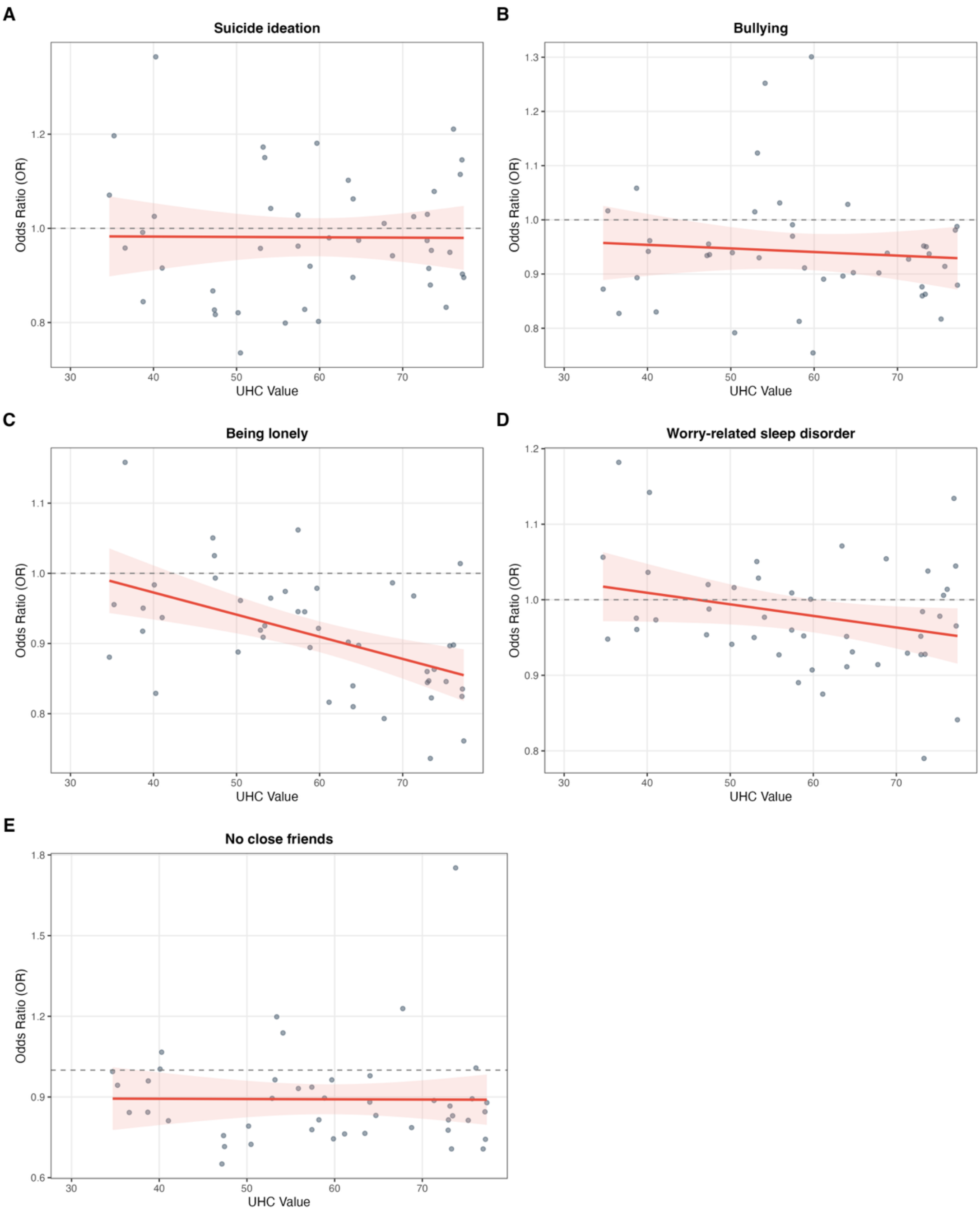

Figure S1. The scatter plot between UHC index and the odds of times of brush teeth per day and mental health outcomes.

OR – odds ratio, UHC – universal health coverage.

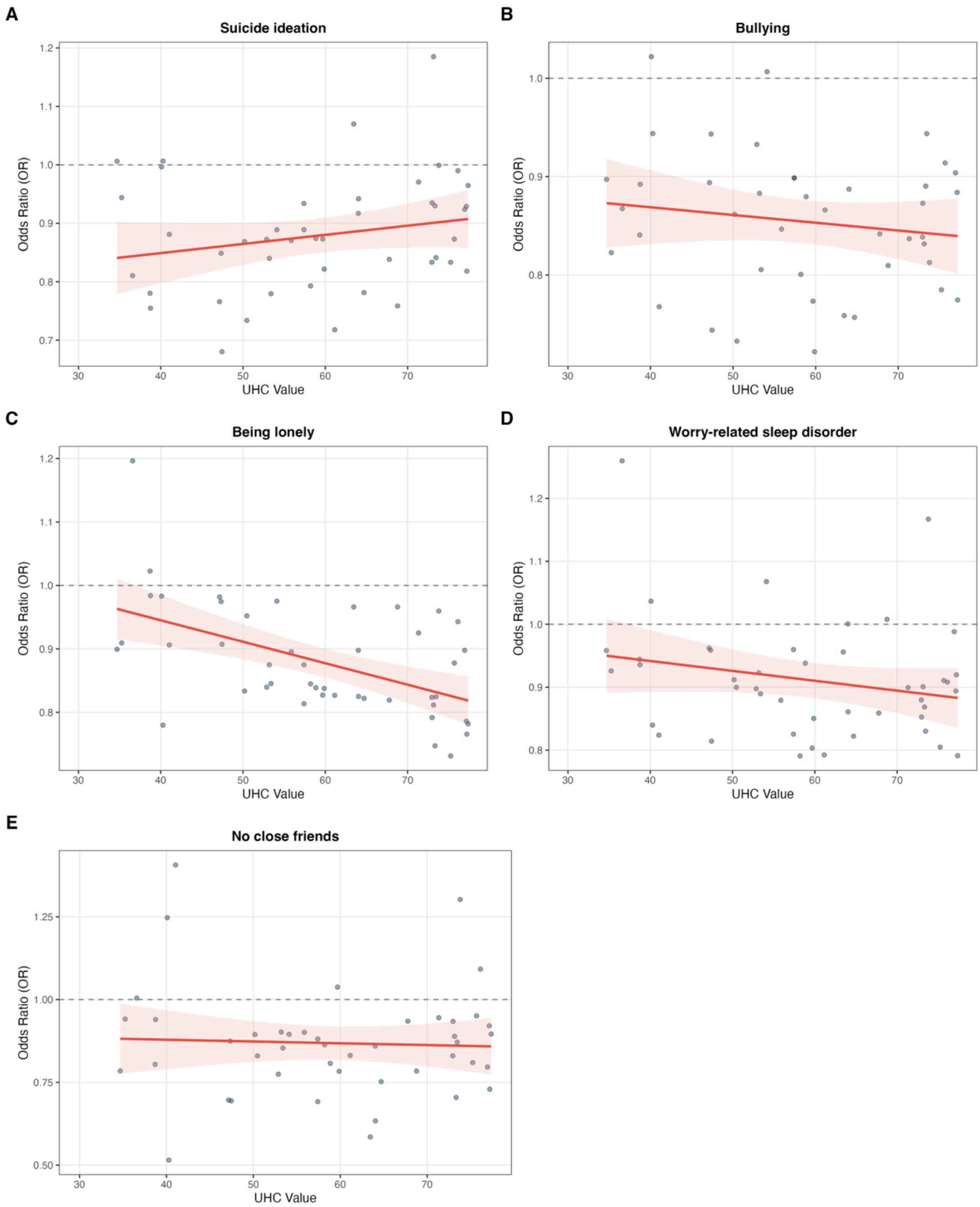

Figure S2. The scatter plot between UHC index and the odds of wash hands before eating and mental health outcomes.

OR – odds ratio, UHC – universal health coverage.

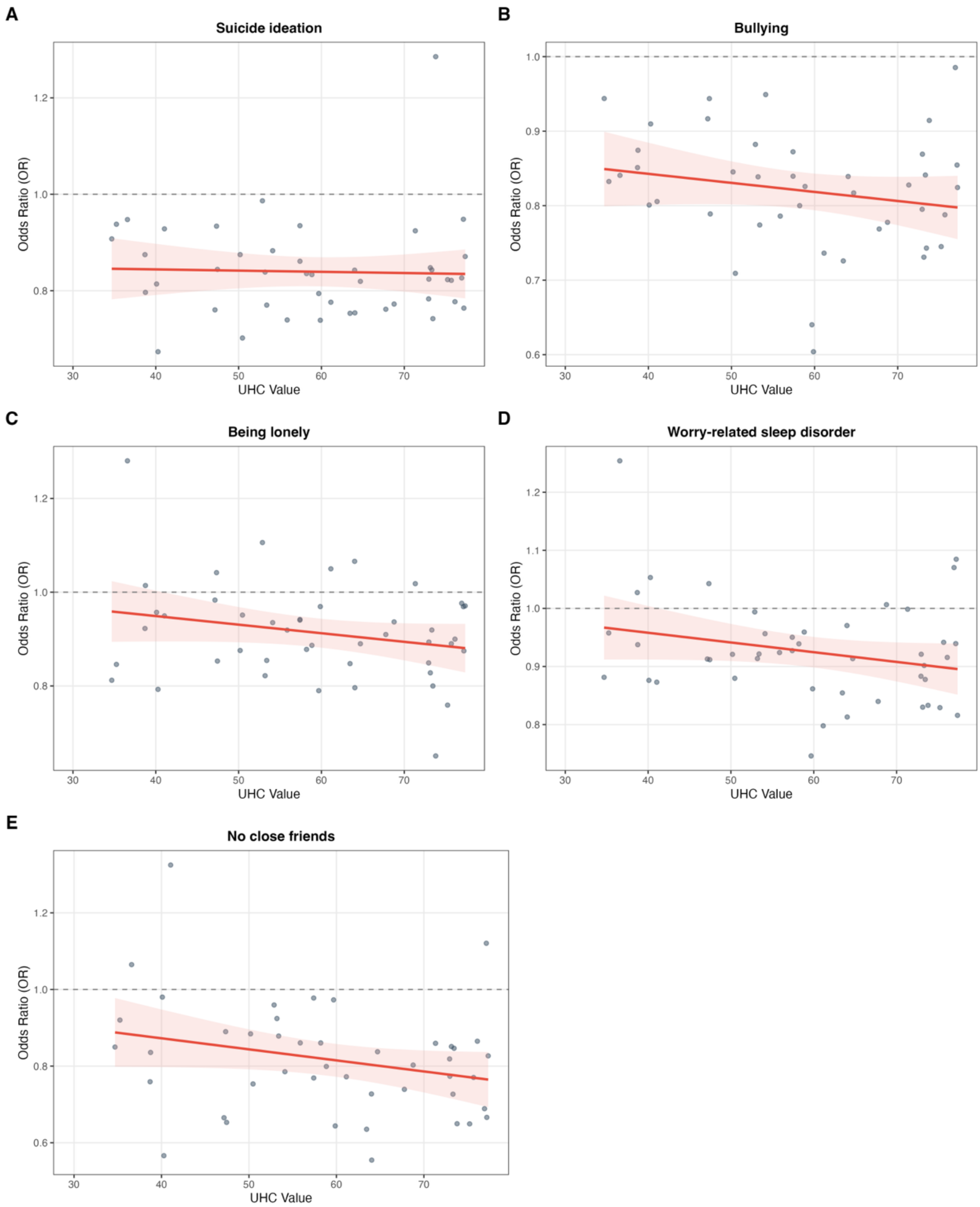

Figure S3. The scatter plot between UHC index and the odds of wash hands after toilet and mental health outcomes.

OR – odds ratio, UHC – universal health coverage.

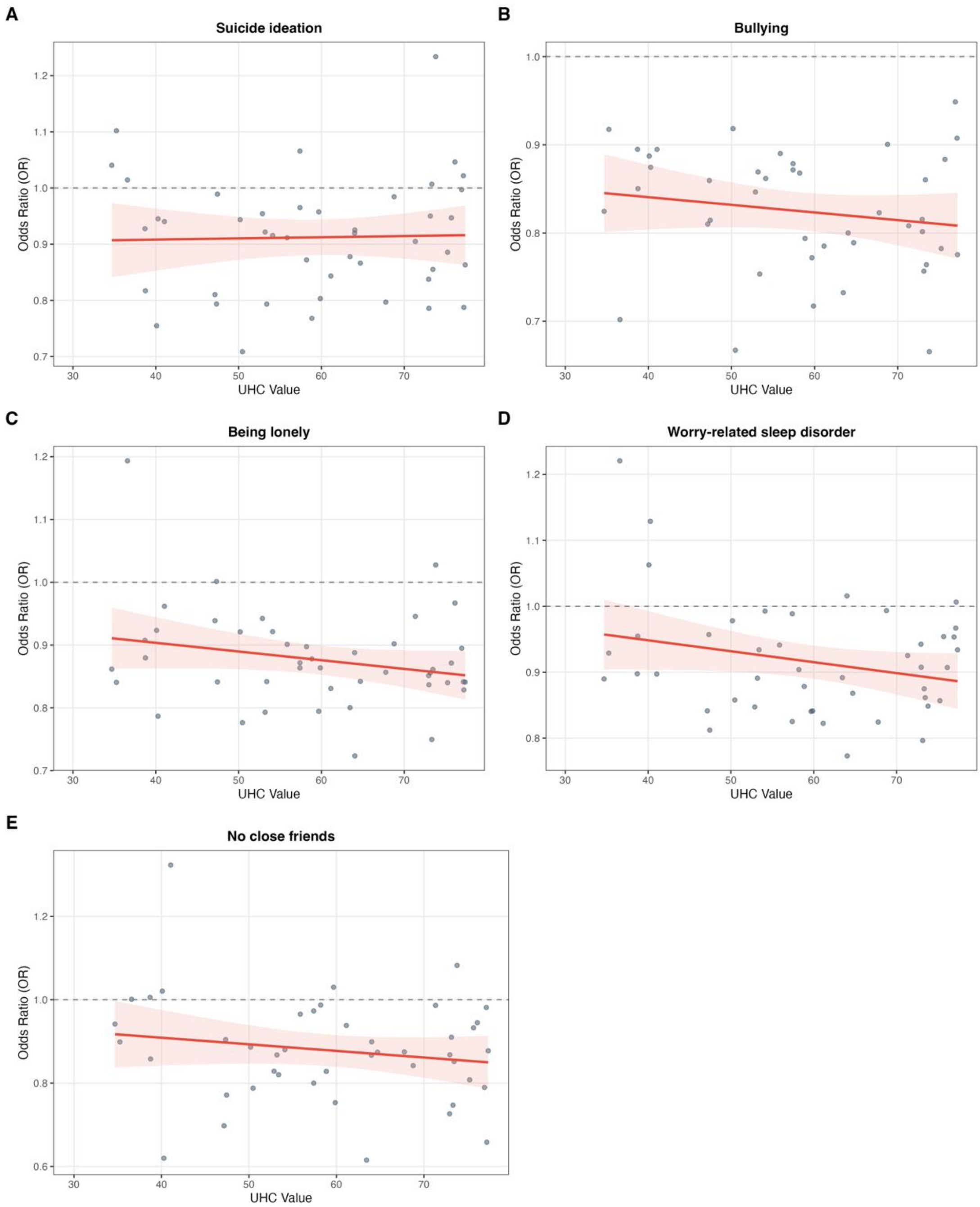

Figure S4. The scatter plot between UHC index and the odds of wash hands with soap and mental health outcomes.

OR – odds ratio, UHC – universal health coverage.

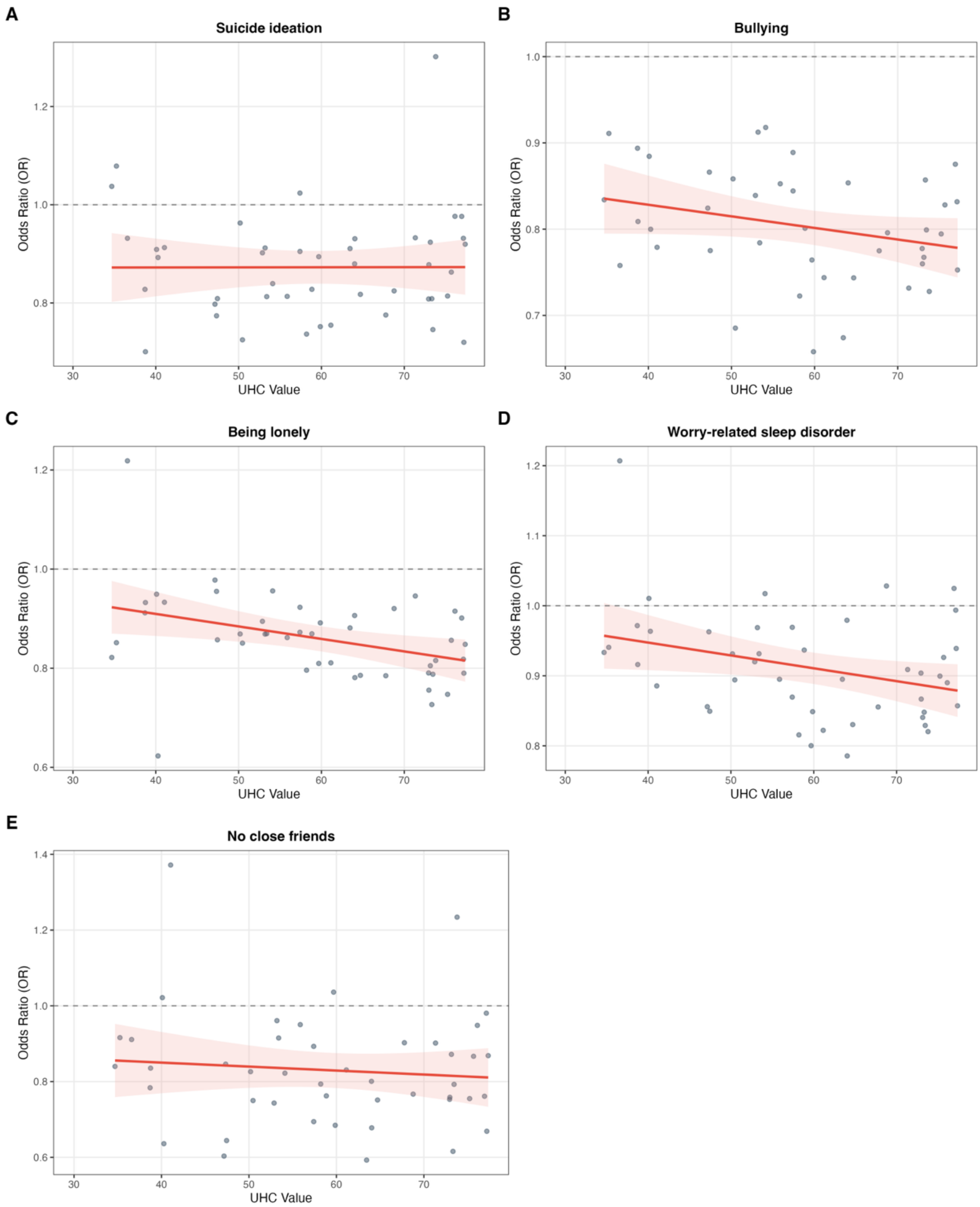

Figure S5. The scatter plot between UHC index and the odds of individual hygiene scores and mental health outcomes.

OR – odds ratio, UHC – universal health coverage.
